# Novel insight into m6A regulator-mediated methylation modification patterns and immune characteristics in intracranial aneurysm

**DOI:** 10.3389/fnagi.2022.973258

**Published:** 2022-08-11

**Authors:** Shifu Li, Qian Zhang, Ling Weng, Ying Han, Jian Li

**Affiliations:** ^1^Department of Neurosurgery, Xiangya Hospital, Central South University, Changsha, China; ^2^Department of Neurology, Xiangya Hospital, Central South University, Changsha, China; ^3^National Clinical Research Center for Geriatric Disorders, Central South University, Changsha, China; ^4^Department of Oral and Maxillofacial Surgery, Center of Stomatology, Xiangya Hospital, Central South University, Changsha, China; ^5^Center for Medical Genetics & Hunan Key Laboratory of Medical Genetics, School of Life Sciences, Central South University, Changsha, China; ^6^Center for Hydrocephalus, Xiangya Hospital, Central South University, Changsha, China

**Keywords:** intracranial aneurysm, m6A regulators, apoptosis, immune infiltrations, signature

## Abstract

**Background:**

Growing evidence demonstrated that m6A modification in cardiovascular diseases. However, how it is involved in the intracranial aneurysm (IA) is still unclear. This study aimed to identify the role of m6A modification in IA.

**Methods:**

Three datasets downloaded from the Gene Expression Omnibus (GEO) database were used, including GSE122897, GSE15629, and GSE3679. The landscapes of 24 m6A regulators were depicted using the STRING database, Pearson’s correlation analysis, and Wilcoxon test. The targets of differentially expressed m6A (DEm6A) were predicted in the m6A2Target database and the modification m6A sites of hub targets were identified in SRAMP online tool. A diagnostic model based on DEm6A was constructed and verified in training and test databases. A consensus clustering algorithm was performed to classify IA patients into distinct m6A-related clusters. Functional analyses including gene ontology, Kyoto Encyclopedia of Genes and Genomes (KEGG), gene set variation analysis, and gene set enrichment analysis analyses were conducted to elucidate the underlying mechanisms. ssGSEA algorithm was performed to uncover the immune characteristics. A PCA method was adopted to quantify the m6A score.

**Results:**

Nine DEm6A (IGF2BP1, IGF2BP3, YTHDF2, ZNF217, RBM15, YTHDF3, YTHDC1, FTO, and LRPPRC) significantly differed between IA and controls. Biological annotations showed that immune-related pathways (such as complement activation, inflammatory response, and interleukin signaling) and apoptosis were more enriched in IAs than in controls. Immune analyses indicate that the abundance of immune cells, immune responses, and HLA gene expression were elevated in IA samples than in controls. PCA results showed that IA has a lower m6A score than controls. An immune/apoptosis-related network modified by DEm6A was constructed. The m6A sites of six hub targets (CDK1, ASPM, AURKB, BUB1B, MKI67, and TPX2) were predicted with very high confidence. A diagnostic model with four genes (LRPPRC, YTHDF3, IGF2BP1, and ZNF217) was constructed and verified. Two m6A modification subtypes were identified with unsupervised cluster analysis. Immune infiltration analysis revealed that cluster 1 had higher immune activation than cluster 2. Further study showed that cluster 1 had a larger proportion of ruptured IAs.

**Conclusion:**

The m6A modification may shape the IAs microenvironment and participates in the formation and rupture of IAs by regulating immune infiltration.

## Introduction

Intracranial aneurysm (IA) is defined as a localized dilation and thinning of cerebral artery walls, which preferentially form at the arterial bifurcation of the circle of Willis. Saccular unruptured intracranial aneurysms (UIAs) have an incidence of 3% in the adult population, and the number of aneurysm cases detected is continuously increasing with the advances of imaging techniques (Etminan and Rinkel, 2016). Subarachnoid hemorrhage, caused by IA rupture, is associated with mortality rates of more than 30% (Petridis et al., 2017) and leads to severe neurological dysfunction and disability. Treatment involves achieving complete IA occlusion while preserving blood flow through parent, branching, and perforating vessels. Surgical clipping and endovascular treatment are the two main techniques for treating IAs. Risk factors, such as a family history of IA, female sex, cigarette smoking, alcohol consumption, inflammation, and high blood pressure, have been found to contribute to the formation, growth, and rupture of IAs (Chalouhi et al., 2013; Bakker et al., 2020). However, the cellular and molecular mechanisms leading to IA formation and rupture have not been fully elucidated.

*N6*-methyladenosine (m6A) methylation is the most prevalent mRNA modification of many eukaryotic species, according to an emerging research field of posttranscriptional gene regulation (Shi et al., 2019). m6A modification exhibits dynamic and reversible changes and is regulated by the interplay between “writers” (methyltransferases), “erasers” (demethylases), and “readers” (methyl-binding proteins). m6A “writers” and “erasers” determine the m6A modification on a specific mRNA, and “readers” can recognize and bind to modification sites and transmit the code to downstream effectors (Yang et al., 2018). Emerging evidence points to the role of RNA m6A modification in cardiovascular diseases, such as coronary heart disease, pulmonary hypertension, cardiac hypertrophy, and heart failure (Wu et al., 2020; Hu et al., 2021). Recent studies have also discovered the potential roles of RNA m6A modification in abdominal aortic aneurysms (Zhong et al., 2020), aortic dissecting aneurysms (Ma et al., 2020), and ischemic stroke (Chang et al., 2022). However, it is still unclear what role m6A plays in the pathophysiology of IAs.

Previous studies have investigated the underlying mechanism of various vascular diseases, such as abdominal aortic aneurysm (Li et al., 2021; Guo et al., 2022) and myocardial infarction (Shi et al., 2021), from the perspective of m6A modification based on the Gene Expression Omnibus (GEO) database. To the best of our knowledge, this is the first study to comprehensively describe and explore the roles of m6A modification in IA pathogenesis. In the present work, we downloaded IA datasets from the GEO database and collected 24 m6A regulators from previous studies. The landscapes of m6A regulators in IAs were depicted, and the differentially expressed m6A regulators (DEm6A) were explored. The m6A targets were predicted from the m6A2Target database, and the m6A modification sites of hub targets were investigated using the SRAMP online database. A diagnostic model based on DEm6A was constructed and validated. An unsupervised cluster analysis based on the expression profile of DEm6A was performed to generate two m6A phenotypes in the IA samples. We also identified m6A phenotype-related markers. Early studies showed the involvement of immune activities in IA development (Hosaka and Hoh, 2014) and high regulation by m6A methylation (Shulman and Stern-Ginossar, 2020). Our work explored the different immune characteristics, such as immune cell infiltration, immune response activity, and human leukocyte antigen (HLA) gene expression, for the IAs and the two m6A phenotypes. A correlation between m6A regulators and immune characteristics was also discovered. The biological functions involved in IAs were elucidated by integrating various algorithms, such as gene set variation analysis (GSVA) and gene set enrichment analysis (GSEA).

## Materials and methods

### Collection and preprocessing of data

**FIGURE 1:**
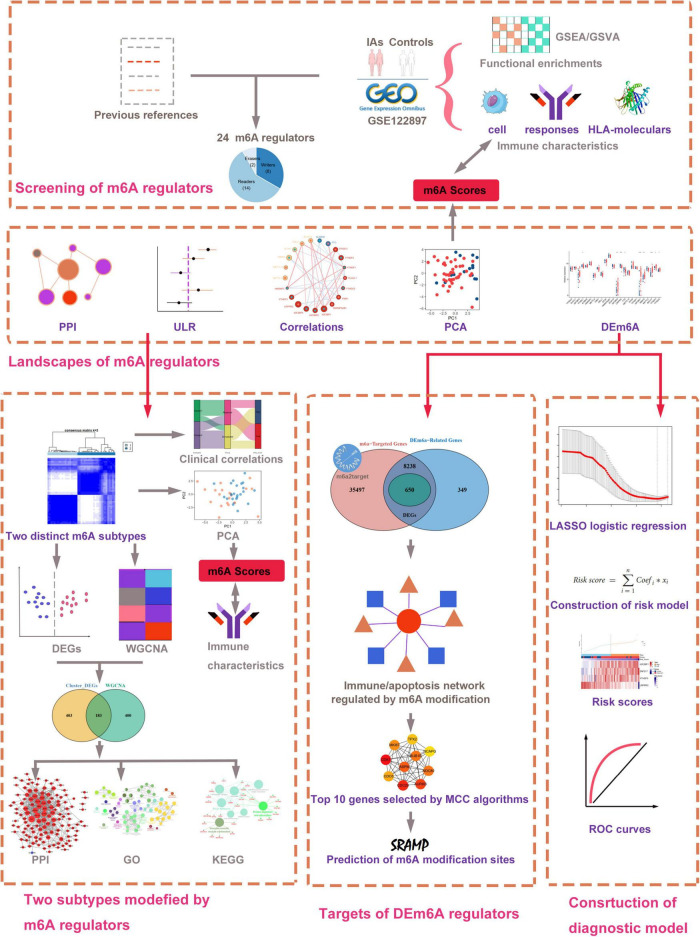
Workflow of data analysis in our present work. DEGs, differentially expressed genes; DEm6A, differentially expressed m6A regulator; ULR, univariable logistic regression; GSEA, gene set enrichment analysis; GSVA, gene set variation analysis; GO, Gene Ontology; KEGG, Kyoto Encyclopedia of Genes and Genomes; LASSO, least absolute shrinkage and selection operator; WGCNA, weighted gene co-expression network analysis; PCA, principal component analysis; PPI, protein–protein interaction.

The research flowchart of this work is displayed in Figure 1. We collected gene expression data of patients with IAs and healthy controls from the GEO database. A total of three datasets were selected in our study, including GSE122897, GSE15629, and GSE36791. GSE122897 contained 44 aneurysms (22 ruptured, 21 unruptured aneurysms, and 1 undetermined aneurysm excluded in this study), and 16 control artery tissues were utilized as the training cohort in the platform of GPL16791 Illumina HiSeq 2500. The GSE15629 dataset (eight ruptured, six unruptured aneurysms, and five control artery tissues) was designated as the training cohort on the GPL6244 Affymetrix Human Gene 1.0 ST Array. To further discover whether this model is useful in clinical practice, we tested our diagnosis model on GSE36791 datasets (platform, GPL10558 Illumina HumanHT-12 V4.0 expression beadchip), an independent peripheral blood dataset. The probe with the maximal expression value was annotated into the homologous gene symbol according to the platform’s annotation information if multiple probes matched one gene.

### Detection and interactions of the m6A RNA methylation regulator

Overall, 24 m6A regulators were selected according to a recent publication (Liu T. et al., 2022), including eight writers (METTL3, METTL4, METTL14, RBM15, WTAP, CBLL1, ZNF217, ZC3H13), two erasers (FTO, ALKBH5), and 14 readers (YTHDC1, YTHDC2, YTHDF1, YTHDF2, YTHDF3, IGF2BP1, IGF2BP2, IGF2BP3, HNRNPA2B1, HNRNPC, FMR1, LRPPRC, ELAVL1, EIF3A). The expression profiles of the 24 m6A regulators were extracted from the GSE122897 datasets. A protein-protein interaction (PPI) network was constructed using the Search Tool for the Retrieval of Interacting Genes database (STRING^1^) to detect potential relationships among the m6A regulator-encoded proteins. Correlation analysis between the m6A regulators was performed using Pearson’s correlation methods. We conducted the Wilcoxon test to identify the significantly differentially expressed m6A regulators (DEm6A) between IA and healthy samples. The expression patterns of DEm6A were visualized with a heatmap.

### Biological annotations for intracranial aneurysms

The gene sets “c5.go.bp.v7.5.1.symbols,” “h.all.v7.5 .1.symbols,” and “c2.cp.kegg.v7.5.1.symbols” were downloaded from the MSigDB database to enrich the biological processes (BP) of gene ontology (GO), HALLMARK pathways, and Kyoto Encyclopedia of Genes and Genomes (KEGG) pathways. The ‘fgsea’ package in R was used to display the enrichment results of GSEA for GO:BP. The GSVA algorithm was used to calculate the HALLMARK and KEGG pathway activation scores, which were applied using the R package “GSVA.” The R package “limma” was used to compare the differences in pathway activation scores between IAs and controls (Ritchie et al., 2015).

### Correlation between m6A regulators and immune characteristics

The abundances of infiltrating immune cells of 28 cell types were evaluated with a single-sample gene set enrichment analysis (ssGSEA) algorithm using the “GSVA” R package. We also conducted ssGSEA to assess the immune response activity and compared the relative expression levels of 21 HLA genes. Pearson’s correlation analysis was used to determine the correlation between m6A regulators and immune characteristics.

### m6A score generation and correlations with immune characteristics in intracranial aneurysms and controls

To quantify the m6A modification level per individual, we established an evaluation index called the m6A score. Principal component analysis (PCA) was conducted based on the expression patterns of the 24 m6A regulators using the prcomp function to assess the distinguishable ability for identified subtypes. PC1 and PC2 were extracted to form signature scores. We then applied a method similar to GGI to construct the m6A score (Sotiriou et al., 2006).


m6A⁢score=∑(P⁢C⁢1⁢i+P⁢C⁢2⁢i)

where i shows the expression of m6A regulators.

The relationships between the m6A score and immune characteristics were also assessed using Pearson’s correlation analysis.

### Correlation analysis between m6A RNA methylation regulators and targets

Three methods were integrated to screen out m6A targets as follows:

(1) The differentially expressed genes (DEGs) between IAs and healthy controls were analyzed using the “limma” R package, with the cutoff criteria of *p* < 0.05 and | log2fold change (FC)| > 1.
(2) Pearson correlation analysis was performed to analyze the relevance between genes and DEm6A in GSE122897. DEm6A-related genes were significantly related to at least one DEm6A (| Pearson’s correlation coefficient | ≥ 0.5 and *p* < 0.05).
(3) The m6A2Target database integrates many validated and potential m6A targets that have been verified through high-throughput analysis of m6A RNA methylation regulators, including RIP-seq, ChIP-seq, RNA-Seq, and m6A-Seq (Deng et al., 2021). The m6A2Target database was applied to predict the DEm6A target genes.

We intersected the DEGs, DEm6A-related genes, and DEm6A-targeted genes based on the three methods to identify the m6A targets modified by DEm6A regulators. We performed GO:BP and KEGG enrichment analyses using the “clusterProfiler” R package.

Considering the pivotal role of the immune system and apoptosis in the pathogenesis of IAs, we collected 2483 immune-related genes and 2747 apoptosis-related genes from the ImmPort (Bhattacharya et al., 2014) and MSigDB databases (Liberzon et al., 2015), respectively. The apoptosis-, immune-, and apoptosis/immune-related genes overlapped with target genes of DEm6A regulators. The interactions between m6A regulators and targets were visualized in Cytoscape software (v3.8.2). A PPI network was prepared for the DEm6A regulators and targets using the STRING database, with a confidence score > 0.4 as the cutoff criterion.

### m6A mechanism between DEm6A regulators and hub targets

We used MCC algorithms in the cytoHubba plugin of Cytoscape to evaluate and identify hub targets. To initially explore whether DEm6A and hub targets could constitute m6A-dependent mechanisms, the SRAMP online database (Zhou et al., 2016)^2^ was applied using either genomic sequences or cDNA sequences as its input. SRAMP was based on three types of random forest classifiers, namely, binary (positional binary encoding of nucleotide sequence), KNN (K-nearest neighbor), and spectrum (nucleotide pair spectrum), and could predict the precise location of m6A modification on mRNA and provide the confidence of every modification site. Only the m6A sites of targets with very high confidence were retained. A coexpression network of these hub targets was constructed using GeneMANIA^3^.

### Development and validation of a m6A diagnostic model

We randomly divided the samples in the GSE122897 dataset into training and testing datasets at a ratio of 6:4. Least absolute shrinkage and selection operator (LASSO) logistic regression was used to further screen out DEm6A using the “glmnet” package. The minimum lambda was set as the optimal value. The risk score for each sample was calculated as follows: Risk scores = Σ(coefficient i * expression of signature gene (i). The area under the curve (AUC) under the receiver operating characteristic (ROC) curve was quantified to predict the model probabilities. The testing dataset was used for internal validation, and the GSE15629 and GSE36791 datasets were used for external validation.

### Unsupervised cluster analysis of m6A regulatory patterns in intracranial aneurysms

Based on the expression profile of the nine DEm6A regulators, we performed an unsupervised cluster analysis to identify distinct m6A subtypes using the R package “ConsensusClusterPlus” (Wilkerson and Hayes, 2010), and the consensus clustering algorithm was run 1000 times to guarantee the robustness of the clustering. The consensus matrix plot, consensus cumulative distribution function (CDF) plot, relative alterations in the area under the CDF curve, and tracking plot were implemented to find the optimal number of clusters.

### Identification of m6A phenotype-related genes

m6A phenotype-related DEGs between two distinct m6A subtypes were selected based on the criteria (*p* < 0.05 and | log2FC| > 1). To further screen out the m6A phenotype-related genes, we constructed a weighted gene coexpression network analysis (WGCNA) to identify m6A phenotype-related coexpression genes using the “WGCNA” R package. The key modules were identified based on the correlation between different modules and subgroups using Pearson’s correlation method. The enrichment of GO and KEGG analyses was conducted in each potential module. The genes with cor.geneModuleMembership > 0.8 (the correlation between the gene and a certain clinical phenotype) and cor.geneTraitSignificance > 0.2 (the correlation between the module eigengene and the gene expression profile) were defined for screening key genes in a module.

We overlapped the m6A phenotype-related DEGs and WGCNA module genes and regarded them as m6A phenotype-related genes. The PPI network of m6A phenotype-related genes was constructed using the STRING database and visualized in Cytoscape software. The ClueGO plugin of Cytoscape was applied to explore the biological mechanisms of m6A phenotype-related genes.

### Biological enrichment analysis and immune infiltration for distinct m6A modification patterns

To elucidate the different biological mechanisms between distinct m6A patterns in IAs, we applied a “GSVA” algorithm as mentioned above to calculate the HALLMARK and KEGG pathway activation scores. The immune characteristics were evaluated using the “ssGSEA” method, and the expression levels of HLA molecules were compared between the two m6A patterns.

### m6A score generation and correlations with clinic and immune characteristics in intracranial aneurysms

Using the PCA method, the generation of m6A scores was based on the expression profiles of 24 m6A regulators in IA samples. The m6A score was compared between two distinct m6A patterns. We further divided the IA samples into high- and low-score groups with the cutoff as the median. The relationships of rupture status, distinct m6A subtypes, and different m6A groups are displayed with a histogram and Sanky diagram. The correlation between the m6A score and immune cells and responses was determined using Pearson’s correlation analysis.

### Patient collection and real-time quantitative polymerase chain reaction

Patients (≥18 years old; IA, *n* = 12) who were diagnosed with IA at Xiangya Hospital of Central South University were recruited for this research between December 2021 and June 2022. Patients with other diseases or complications were excluded. In total, 12 healthy controls who were gender- and age-matched were also selected.

Peripheral blood monocytes (PBMCs) were isolated from the blood samples of IA and healthy persons using Ficoll solution (Solarbio, Beijing, China). Total RNA was extracted from the PBMCs using TRIzol reagent (Takara, Kyoto, Japan) according to the manufacturer’s protocol. Then, reverse transcription reactions were performed using 500 ng RNA and an Evo M-MLV reverse transcriptase kit (Accurate Biotechnology, Hunan, China) according to the manufacturer’s instructions. A SYBR Green Pro Taq HS Kit (Accurate Biotechnology, Hunan, China) and 0.4 μmol of each primer pair were used to amplify the cDNA, which was then evaluated in an ABI 7500 real-time PCR system (Applied Bioscience, Foster City, CA, United States). The results were analyzed using the 2^–ΔΔCt^ method and expressed as the ratio of the internal control, GAPDH. The primer sequences used for RT–qPCR are available in Supplementary Table 1.

### Statistics analyses

All statistical analyses were performed using R software (version R-4.1.0). The Wilcoxon test was used for statistical analysis between the two groups. The proportion of clinical traits in different groups was compared with the chi-square test. The relationships of genes with genes and immune cells were constructed using Pearson’s correlation method. LASSO regression analysis was carried out using the “glmnet” package. Unsupervised cluster analysis was performed using the R package “ConsensusClusterPlus.” *p* < 0.05 indicated statistical significance. The significance level was denoted as follows: *p < 0.05, **p < 0.01, and ***p < 0.001.

## Results

### The landscape of m6A regulators between healthy and intracranial aneurysm samples

**FIGURE 2:**
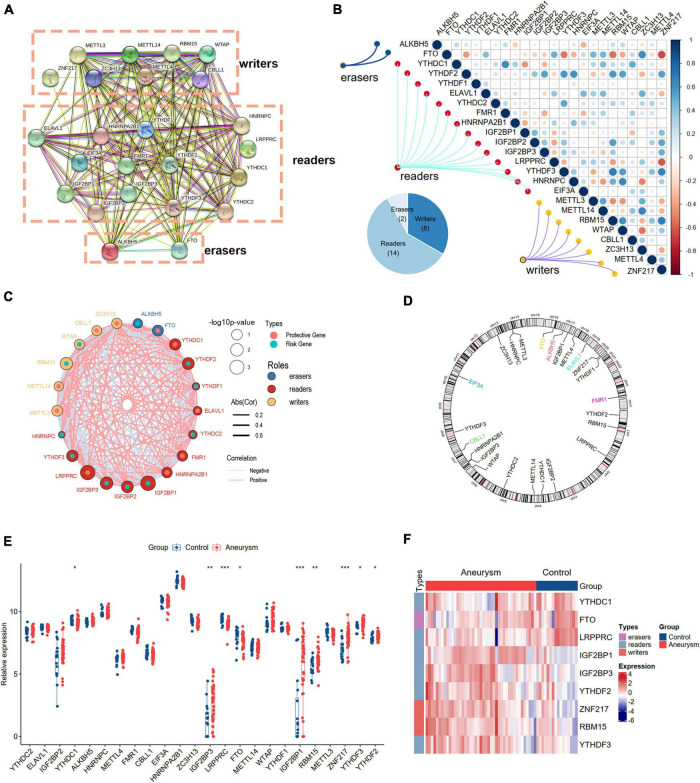
Landscape of m6A RNA methylation regulators in IA. **(A)** PPI network of 24 m6A regulators. **(B)** Correlation heat map of 24 m6A regulators. The positive correlation was marked with blue, and negative correlation was marked with red. **(C)** The interactions between m6A regulators and their diagnostic value using univariable logistic regression. The circle size indicates the *p*-value of the univariable logistic regression, and the lines linking the m6A regulators indicate their interactions. **(D)** Circus plot of chromosome distributions of the 24 m6A regulators. **(E)** The expression difference of the 24 m6A regulators between IA and controls. **(F)** A heat map showing the expression patterns of nine differently expressed m6A genes. Significance level was denoted by *p-value < 0.05, **p-value < 0.01, ***p-value < 0.001.

We curated and analyzed a set of 24 acknowledged m6A regulators (14 writers, 8 readers, and 2 erasers; Table 1). The PPI network revealed the regulatory interactions between these m6A regulators (Figure 2A). To explore the association between different m6A regulators, we investigated the transcriptome relationships and found a close correlation among writers, readers, and erasers (Figures 2B,C). We also located the positions of the 24 m6A regulators on their chromosomes (Figure 2D). Expression profiling revealed that the expression levels of nine m6A regulators, designated DEm6A, significantly differed between IAs and controls (Figure 2E). Six genes (*IGF2BP1*, *IGF2BP3*, *YTHDF2*, *ZNF217*, *RBM15*, and *YTHDF3*) were upregulated, and three genes (*YTHDC1*, *FTO*, and *LRPPRC*) were downregulated. The expression patterns of the nine m6A regulators are depicted with a heatmap (Figure 2F). In addition, a forest plot shows the univariable logistic regression results of DEm6A (Supplementary Figure 1).

**TABLE 1:** The detailed descriptions of 24 m6A regulators.

Gene	Gene description	Chr	Role
ALKBH5	alkB homolog 5, RNA demethylase	17	Erasers
FTO	FTO alpha-ketoglutarate dependent dioxygenase	16	Erasers
YTHDC1	YTH domain containing 1	4	Readers
YTHDF2	YTH *N6*-methyladenosine RNA binding protein 2	1	Readers
YTHDF1	YTH *N6*-methyladenosine RNA binding protein 1	20	Readers
ELAVL1	ELAV like RNA binding protein 1	19	Readers
YTHDC2	YTH domain containing 2	5	Readers
FMR1	FMRP translational regulator 1	X	Readers
HNRNPA2B1	Heterogeneous nuclear ribonucleoprotein A2/B1	7	Readers
IGF2BP1	Insulin like growth factor 2 mRNA binding protein 1	17	Readers
IGF2BP2	Insulin like growth factor 2 mRNA binding protein 2	3	Readers
IGF2BP3	Insulin like growth factor 2 mRNA binding protein 3	7	Readers
LRPPRC	Leucine rich pentatricopeptide repeat containing	2	Readers
YTHDF3	YTH N6-methyladenosine RNA binding protein 3	8	Readers
HNRNPC	Heterogeneous nuclear ribonucleoprotein C	14	Readers
EIF3A	Eukaryotic translation initiation factor 3 subunit A	10	Readers
METTL3	Methyltransferase 3, *N6*-adenosine-methyltransferase complex catalytic subunit	14	Writers
METTL14	Methyltransferase 14, *N6*-adenosine-methyltransferase subunit	4	Writers
RBM15	RNA binding motif protein 15	1	Writers
WTAP	WT1 associated protein	6	Writers
CBLL1	Cbl proto-oncogene like 1	7	Writers
ZC3H13	Zinc finger CCCH-type containing 13	13	Writers
METTL4	Methyltransferase 4, *N6*-adenosine	18	Writers
ZNF217	Zinc finger protein 217	20	Writers

### Biological functions involved in intracranial aneurysms

**FIGURE 3:**
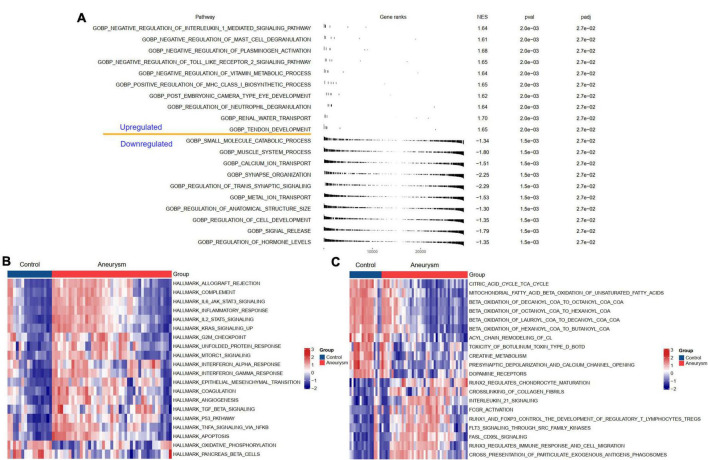
Biological functions and pathways enriched in IAs. **(A)** Up- or down-regulated GO:BP of GSEA results in IA patients relative to controls. **(B)** The GSVA results indicating the top 20 different hallmark pathways between IA and controls. **(C)** The GSVA results indicating the top 20 different KEGG pathways between IA and controls.

To understand the potential biological processes and pathways in the pathogenesis of IAs, we performed GSEA and GSVA to identify the dysregulated GO:BP, HALLMARK, and KEGG pathways in IAs compared to controls. The BP results indicated that, compared with the controls, regulation of neutrophil degranulation, positive regulation of MHC class I biosynthetic process, and negative regulation of plasminogen activation were upregulated in IAs. In contrast, the muscle system process and regulation of transsynaptic signaling were downregulated (Figure 3A). HALLMARK and KEGG analyses showed that immune-related pathways (such as complement activation, inflammatory response, and interleukin signaling) and apoptosis were more active in IAs than controls (Figures 3B,C).

### m6A regulators are related to the immune microenvironment in intracranial aneurysms

**FIGURE 4:**
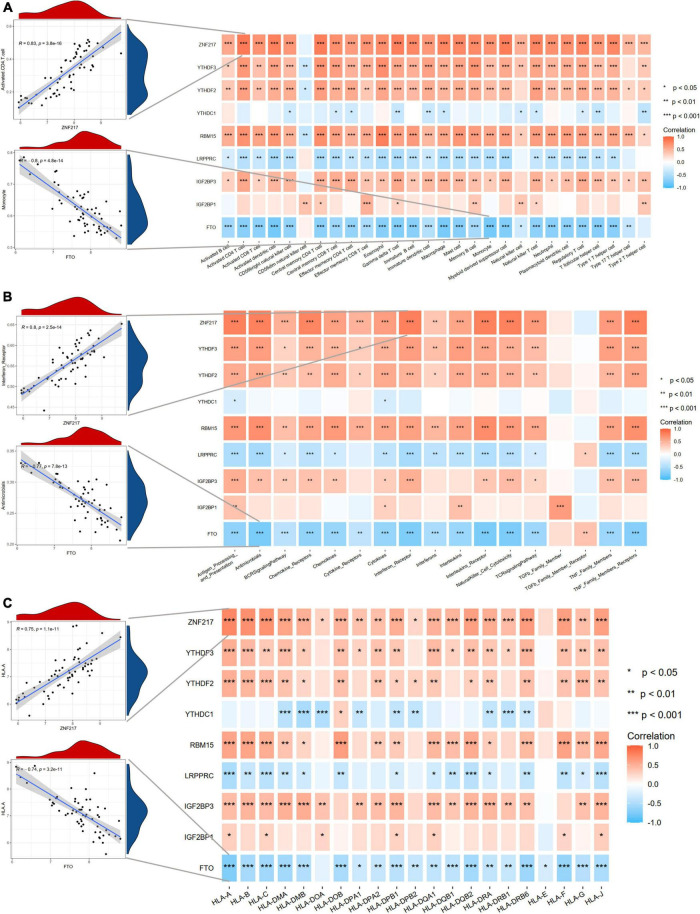
Correlation analyses between m6A regulators and immune characteristics. **(A)** The correlations between DEm6A regulators and immune cells. **(B)** The correlations between DEm6A regulators and immune responses. **(C)** The correlations between DEm6A regulators and HLA genes. The left panel depicted the most positive and negative correlations with scatterplots.

Considering the activated immune function in IA tissue, we further explored the immune characteristics in IAs using the ssGSEA method, including immune cell infiltration, immune responses, and expression levels of HLA molecules. The differences in the abundance of 28 immune microenvironment infiltrating cells, activities of 17 immune responses, and expression levels of 21 HLAs between healthy and IA samples were revealed (Supplementary Figure 2). We observed that most immune characteristics were enhanced in IAs compared to controls. These findings suggested that inflammatory responses were involved in the pathogenesis of IAs. Therefore, we analyzed the correlations between m6A regulators and immune characteristics (Figure 4). The results indicated that downregulated m6A regulators were negatively correlated with immune characteristics, and upregulated m6A regulators were positively correlated with immune characteristics, indicating that immune dysregulation in IAs is affected by m6A RNA methylation. The most positive gene-cell pair was activated CD4 T cells and ZNF217 (*R* = 0.83, *p* < 0.001), whereas the most negative pair was monocytes and FTO (*R* = –0.8, *p* < 0.001). We found that ZNF217 was most positively correlated with interferon receptors (*R* = 0.8, *p* < 0.001), and FTO was most negatively correlated with antimicrobials (*R* = –0.877, *p* < 0.001) from the perspective of immune responses. Among the correlations between m6A regulators and HLA molecules, HLA-A was most positively and negatively correlated with ZNF217 (*R* = 0.75, *p* < 0.001) and FTO (*R* = –0.74, *p* < 0.001), respectively. The above results suggested that m6A regulators may play a pivotal role in the molecular traits and immune infiltration in IAs. ZNF217 and FTO may act as the most important m6A regulators shaping the immune microenvironment.

### The m6A score is associated with immune characteristics in IAs and controls

**FIGURE 5:**
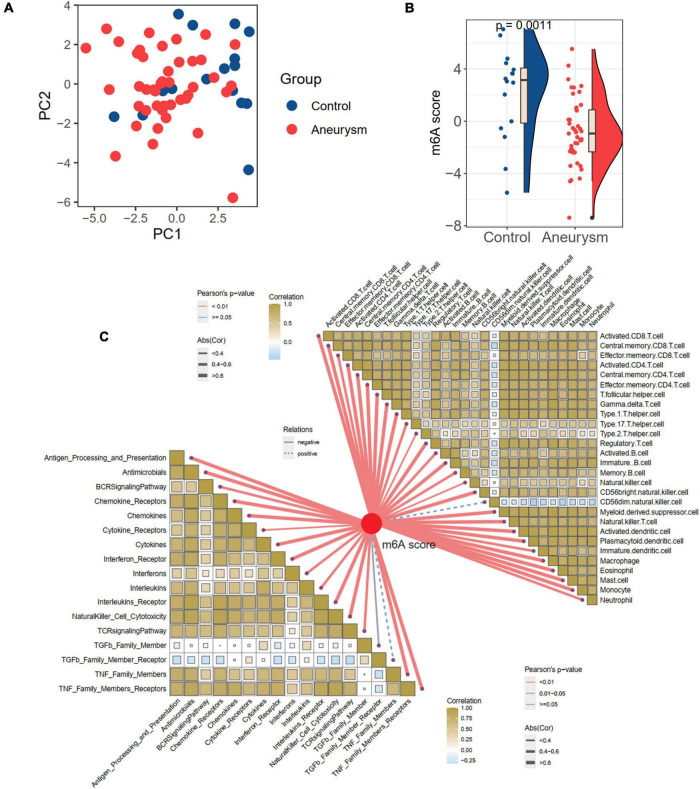
Generation of m6A score and correlation with immune cells and immune responses. **(A)** PCA plot based on the expression profiles of the 24 m6A regulators. **(B)** The difference of m6A score between IA and controls. **(C)** The correlations between m6A score with immune cells (the upper right part) and immune responses (lower left part).

Considering the individual heterogeneity of m6A modification between controls and IAs, we defined an indicator, the m6A score, to comprehensively quantify the m6A modification of each individual. Based on the expression profile of 24 m6A regulators, the diseases and controls were distinguishable by PCA (Figure 5A and Supplementary Table 2). Further analysis revealed a higher m6A score in controls than in IAs (*p* = 0.001; Figure 5B). To explore the relationships between the m6A score and immune characteristics, Pearson’s correlation analysis was performed between the m6A score and immune cells and responses (Figure 5C). The m6A score was significantly negatively related to most immune characteristics. Combined with the results showing that the immune infiltration of IAs was higher than that of the controls, it was inferred that the m6A score is a protective factor in IAs by suppressing inflammatory activities.

### Identification of m6A targets and m6A mechanism for hub targets

**FIGURE 6:**
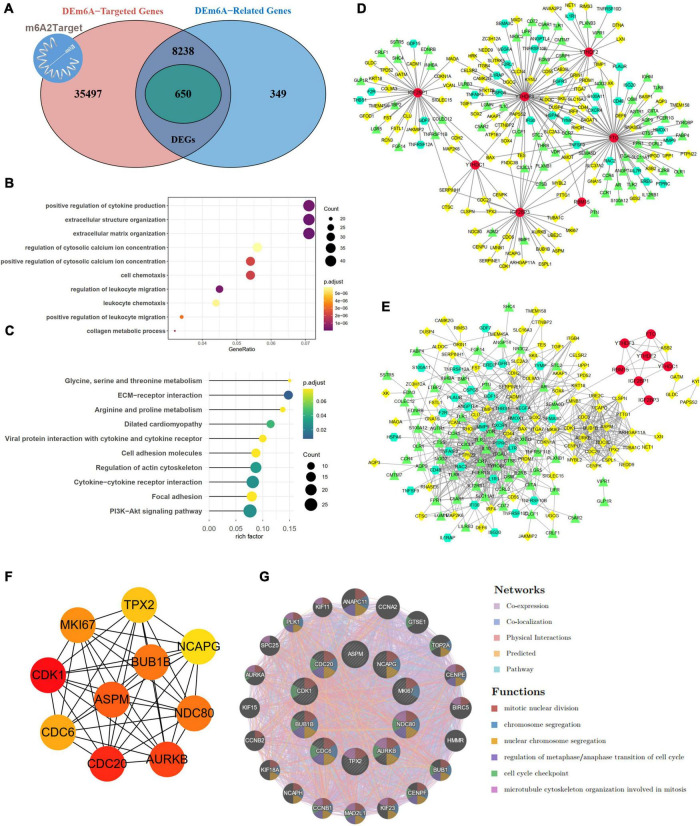
Targets and apoptosis/immune-related genes modified by DEm6A regulators. **(A)** Screening out the 650 potential targets modified by DEm6A regulators in IAs by overlapping the DEGs between IA and controls, DEm6A-related genes, and Dem6A-targeted genes in m6A2Target database. **(B)** Biological processes of Gene Ontology of the DEm6A regulators targets. **(C)** KEGG pathway of the DEm6A regulators targets. **(D)** Association between apoptosis/immune-related genes and DEm6A regulators. Red color represents m6A regulators, green color represents immune-related genes, yellow color represents apoptosis-related genes, and turquoise color represents immune and apoptosis-related genes. **(E)** A PPI network constructed by apoptosis/immune-related genes and DEm6A regulators. **(F)** The top ten hub targets identified by MCC algorithm in CytoHubba. **(G)** Co-expression network of the ten hub targets.

By integrating genes identified with three steps, including DEGs between IAs and controls, DEm6A target genes derived from the m6A2Target database, and genes closely correlated with DEm6A, 650 overlapping targets were identified (Figure 6A). Functional enrichment analysis indicated that these genes were involved in immune-related processes and organization of the extracellular matrix (Figures 6B,C and Tables 2, 3). Because of the crucial roles of immune function and apoptosis, we further screened immune- and apoptosis-related genes from the 650 targets. Overall, 61 immune-related genes (green), 98 apoptosis-related genes (yellow), and 31 immune- and apoptosis-related genes (turquoise) were identified (Supplementary Table 3), and the correlation between m6A regulators and targets was depicted through an interaction network (Figure 6D). A PPI network was constructed in the STRING database and visualized in Cytoscape (Figure 6E) to study their molecular interactions. To identify the hub targets, the top 10 genes were identified using the MCC algorithm of cytoHubba (Figure 6F). The SRAMP online database was applied to find the confidence of every modification site of the hub targets. Six targets (CDK1, ASPM, AURKB, BUB1B, MKI67, and TPX2) were predicted for the m6A sites with very high confidence (Supplementary Figure 3 and Supplementary Table 4). A coexpression network was constructed in which 20 genes were found to interact with the 10 hub targets (Figure 6G). In the complex PPI network, the interaction of the coexpression accounted for 66.16%, colocalization accounted for 16.17%, physical interactions accounted for 10.55%, predicted as 6.63%, and pathway accounted for 0.49%. Functional annotation showed that these genes were involved in the cell cycle.

**TABLE 2:** The biological processes of GO for targets modified by m6A regulators in IA.

ID	Description	BgRatio	p value	p.adjust	q value
GO:0030198	Extracellular matrix organization	393/18862	4.71E-12	9.64E-09	7.47E-09
GO:0043062	Extracellular structure organization	394/18862	5.11E-12	9.64E-09	7.47E-09
GO:0001819	Positive regulation of cytokine production	437/18862	1.41E-10	1.69E-07	1.31E-07
GO:0002685	Regulation of leukocyte migration	205/18862	3.33E-10	3.19E-07	2.47E-07
GO:0032963	Collagen metabolic process	109/18862	1.25E-09	1.00E-06	7.75E-07
GO:0060326	Cell chemotaxis	306/18862	2.98E-09	2.04E-06	1.58E-06
GO:0007204	Positive regulation of cytosolic calcium ion concentration	310/18862	4.10E-09	2.19E-06	1.69E-06
GO:0002687	Positive regulation of leukocyte migration	133/18862	6.65E-09	3.19E-06	2.47E-06
GO:0030595	Leukocyte chemotaxis	226/18862	1.29E-08	5.62E-06	4.36E-06
GO:0051480	Regulation of cytosolic calcium ion concentration	344/18862	1.45E-08	5.81E-06	4.50E-06

**TABLE 3:** The KEGG results for targets modified by m6A regulators in IA.

ID	Description	BgRatio	p value	p.adjust	q value
hsa04512	ECM-receptor interaction	88/8105	2.73E-05	7.77E-03	6.80E-03
hsa04060	Cytokine-cytokine receptor interaction	295/8105	3.93E-04	2.74E-02	2.40E-02
hsa04151	PI3K-Akt signaling pathway	354/8105	4.81E-04	2.74E-02	2.40E-02
hsa04810	Regulation of actin cytoskeleton	218/8105	6.90E-04	3.28E-02	2.87E-02
hsa05414	Dilated cardiomyopathy	96/8105	1.07E-03	4.34E-02	3.80E-02
hsa00330	Arginine and proline metabolism	51/8105	3.10E-03	7.71E-02	6.75E-02
hsa00260	Glycine, serine and threonine metabolism	40/8105	3.88E-03	7.71E-02	6.75E-02
hsa04510	Focal adhesion	201/8105	4.47E-03	7.71E-02	6.75E-02
hsa04514	Cell adhesion molecules	149/8105	4.67E-03	7.71E-02	6.75E-02
hsa04061	Viral protein interaction with cytokine and cytokine receptor	100/8105	4.87E-03	7.71E-02	6.75E-02

### Construction and validation of a m6A regulator diagnostic model

**FIGURE 7:**
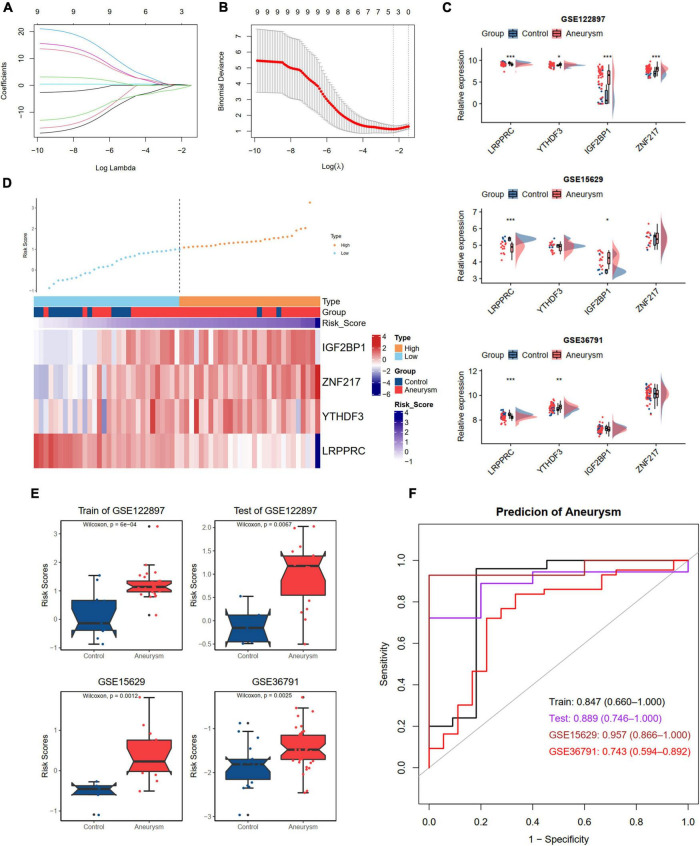
Development and validation an m6A regulators diagnostic model to distinguish IA and controls. **(A,B)** LASSO logistic regression algorithm to screen diagnostic markers in train dataset of GSE122897. **(A)** The coefficients change of different genes with different lambda. **(B)** Cross-validation for tuning parameter selection in the LASSO model. **(C)** The expression levels of four selected DEm6A regulators in GSE122897, GSE15629, and GSE36791 dataset. **(D)** The expression patterns of four selected DEm6A regulators according to the distribution of risk score in the train dataset of GSE122897. **(E)** Comparison of risk score between IA and controls in the training and validation datasets. **(F)** Receiver operating characteristic (ROC) curves predicting IA in the training and validation datasets. Significance level was denoted by *p-value < 0.05, **p-value < 0.01, ***p-value < 0.001.

Developing an accurate diagnostic model to detect IAs in individuals is essential. We divided the GSE122897 dataset into training and test cohorts at a ratio of 6:4. To further screen the DEm6A regulator candidates to construct a diagnostic model, these nine DEm6A were enrolled for LASSO logistic regression analysis in the GSE122897 training set. The calculated regression coefficient and the tenfold cross-validation for tuning parameter selection in the LASSO regression are displayed in Figures 7A,B, respectively. A four m6A regulator diagnostic model was constructed, with the risk scores as follows: Risk score = 3.7166 + (–1.4645 * LRPPRC) + (0.1145 * YTHDF3) + (0.2749 * IGF2BP1) + (0.3802 * ZNF217). The expression levels of the four genes were validated in the GSE15629 and GSE36791 datasets (Figure 7C). Furthermore, we detected the expression levels of the four hub m6A regulators in our clinical blood samples. The results indicated that the expression of LRPPRC and YTHDF3 was significantly different between IAs and controls, whereas no significant difference was observed for IGFBP1 and ZNF217 (Supplementary Figure 4). With the cutoff as the median for the risk score, the individuals were classified into high- and low-risk groups. According to the risk score distributions, the expression patterns of the four m6A regulators in the training set were visualized with a heatmap (Figure 7D). Moreover, we calculated the risk score according to the formula in the test set GSE122897 and the external datasets GSE15629 and GSE36791 (Figure 7E). The diagnostic ability of the risk score was evaluated with ROC curves, indicating that the risk scores had good diagnostic ability in discriminating aneurysms from the control samples, with an AUC of 0.847 (95% CI 0.660–1.000) in the training set, 0.889 (95% CI 0.746–1.000) in the test set, 0.957 (95% CI 0.866–1.000) in the GSE15629 dataset, and 0.743 (95% CI 0.594–0.892) in the GSE36791 dataset (Figure 7F).

### Identification of m6A RNA methylation phenotypes and phenotype-related genes

**FIGURE 8:**
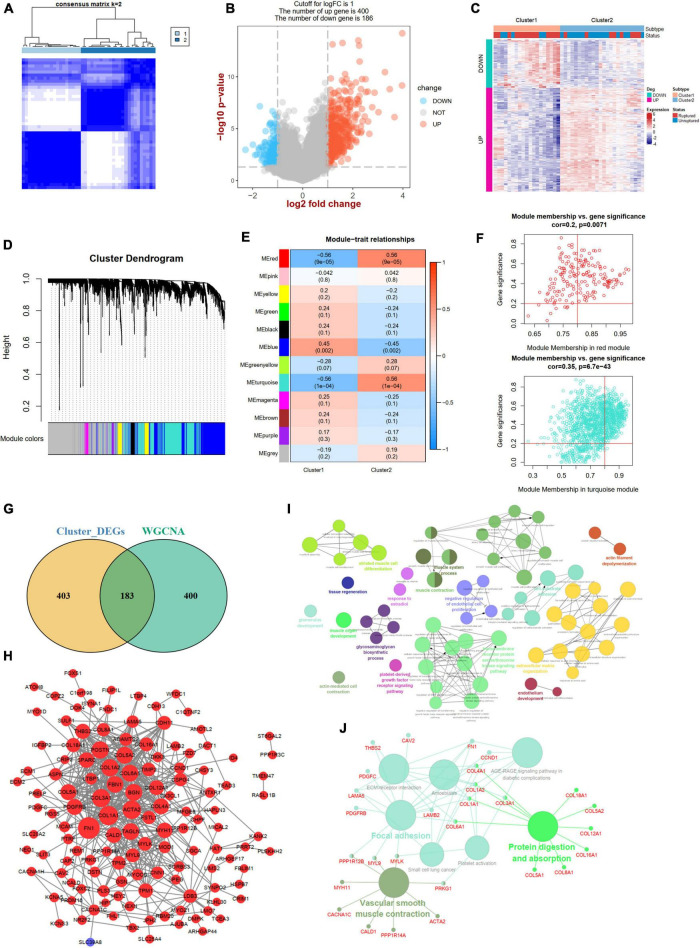
Identification of two m6A modification phenotypes and phenotype-Related Genes. **(A)** Consensus clustering matrix for *k* = 2. **(B)** A volcano plot showing the DEGs between two subtypes. **(C)** A heat map showing the expression patterns of DEGs between two subtypes. **(D)** Gene dendrogram obtained by average linkage hierarchical clustering. **(E)** Heatmap of the correlation between module eigengenes and the two m6A methylation phenotypes. **(F)** A scatterplot of gene significance (GS) for cluster-2 vs. module membership (MM) in the red and turquoise module. **(G)** A Venn plot identifying phenotype-related genes by intersecting DEGs between two subtypes and WGCNA module genes. **(H)** A PPI network of phenotype-related genes. Red color represents up-regulated genes and blue color represents down-regulated genes. **(I,J)** Showing the GO: BP and KEGG enrichment results of phenotype-related genes in ClueGo. DEG, differential expressed genes.

A consistent unsupervised methodology was applied to obtain different clusters for subsequent analysis. The consensus matrix showed that the unsupervised algorithm based on the DEm6A regulators could clearly distinguish the samples into two subtypes (Figure 8A). The consensus CDF plot for k (2–10), relative alterations in the area under the CDF curve, and tracking plot were implemented to find the optimal number of clusters (Supplementary Figures 5A–C). Given the consensus matrix for the analysis, *k* = 2 appeared to be the best choice. In total, 586 DEGs (400 upregulated and 186 downregulated) were identified between the two m6A patterns (Figures 8B,C). To further screen out the m6A phenotype-related genes, a WGCNA algorithm was employed (Figure 8D and Supplementary Figures 6A,B). The module-trait relationship heatmap indicated that the red and turquoise modules correlated most with the two phenotypes (*r* = 0.56; Figure 8E). We also performed GO and KEGG enrichment analyses for the two modules (Supplementary Figures 6C–F). According to the criteria (module membership > 0.8 and gene significance > 0.2), 583 module genes were screened (Figure 8F). By overlapping the DEGs and module genes, we intersected 183 m6A phenotype-related genes (Figure 8G). The PPI network showed close interactions among genes (Figure 8H). The functional annotations revealed that these genes were involved in vascular smooth muscle cells, endothelial cell function, and extracellular matrix organization (Figures 8I,J).

### Biological enrichment analysis and immune infiltration for distinct m6A modification subtypes

**FIGURE 9:**
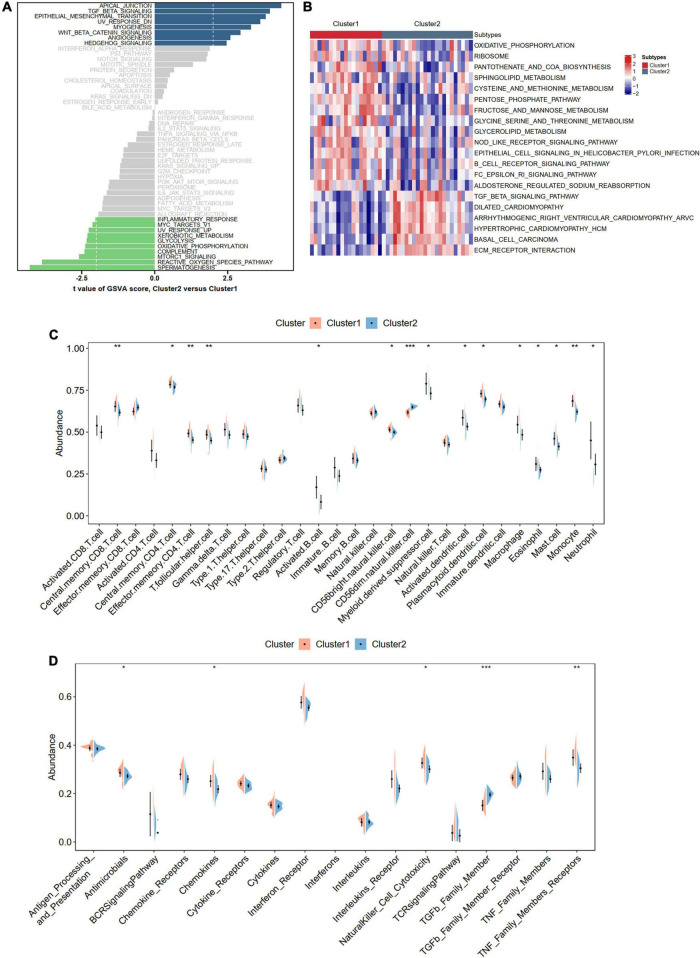
Biological enrichment analysis and immune infiltration for distinct m6A modification phenotypes. **(A,B)** Revealed the Hallmark and KEGG pathways in two different m6A modification phenotypes. **(C)** Violin diagram of the proportion of 28 types of immune cells using ssGSEA algorithm. **(D)** Violin diagram of the proportion of 17 types of immune responses using ssGSEA algorithm. Significance level was denoted by *p-value < 0.05, **p-value < 0.01, ***p-value < 0.001.

To better understand the different biological functions between distinct m6A patterns, we conducted GSVA to identify distinct HALLMARK and KEGG biological pathways. In HALLMARK analysis, we found that Cluster 2 was enriched in apical junction and TGF beta signaling, whereas the inflammatory responses were involved in Cluster 1 (Figure 9A). Consistent with the HALLMARK analysis, the KEGG results also showed that Cluster 2 had a higher score of ECM-receptor interaction and TGF beta signaling but a lower score of immune-related pathways than Cluster 1 (Figure 9B). To further determine the immune characteristics of the two m6A subtypes, we compared the abundance of infiltrating immune cells, activity of the immune response, and expression levels of HLA genes. Unsurprisingly, we observed that Cluster 1 had more immune cell infiltration and immune response and upregulated HLA gene expression (Figures 9C,D and Supplementary Figure 7).

### m6A score generation and correlations with the clinical and immune characteristics in distinct subtypes

**FIGURE 10:**
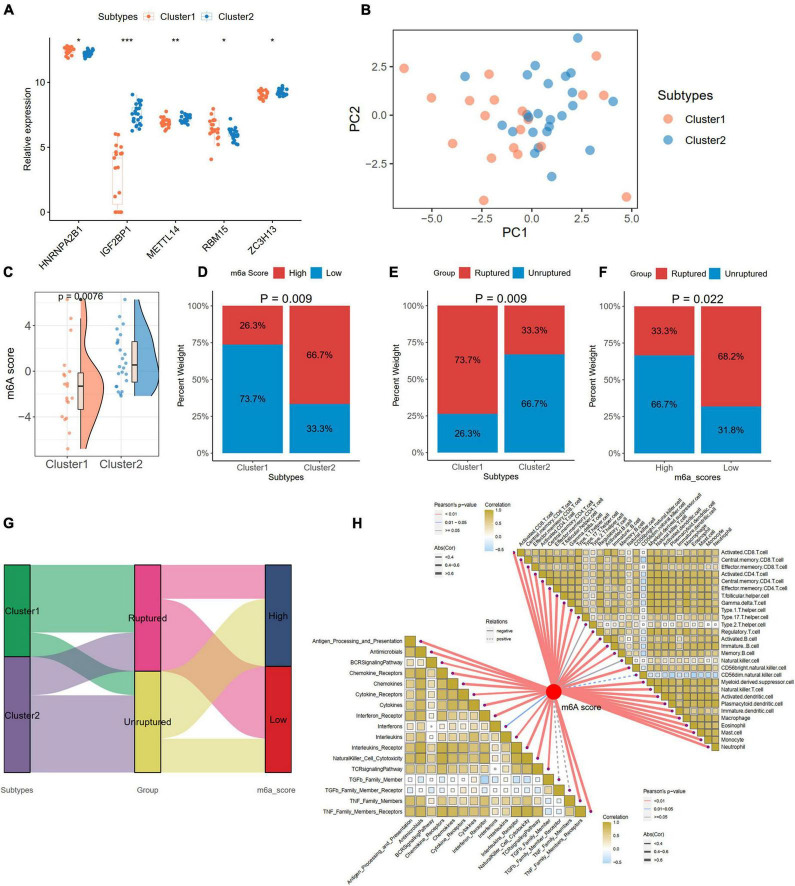
Generation of m6A Score and correlations with clinic and immune characteristics in distinct subtypes. **(A)** Identification of five significant expressed m6A regulator in two subtypes. **(B)** A PCA plot based on the 24 m6A regulators in IA samples. **(C)** Comparison of m6A score from PCA algorithm between two subtypes. **(D)** The percentage of high- and low-m6A score groups in two subtypes. **(E)** The percentage of ruptured IA in two subtypes. **(F)** The percentage of ruptured IA in high- and low-m6A score groups. **(G)** A Sankey diagram indicating the the clinical and molecular correlations. **(H)** The correlations between m6A score with immune cells (the upper right part) and immune responses (lower left part). Significance level was denoted by *p-value < 0.05, **p-value < 0.01, ***p-value < 0.001.

We compared the expression levels of 24 m6A regulators between the two subtypes and found five significantly differentially expressed genes (*HNRNPA2B1*, *IGF2BP1*, *METTL14*, *RBM15*, and *ZC3H13*) between Cluster 1 and Cluster 2 (Figure 10A). PCA based on the 24 m6A regulators indicated that the two subtypes could be distinguished (Figure 10B). We also calculated the m6A score for each IA sample according to the PCA results. We found that Cluster 2 had a higher m6A score than Cluster 1 (Figure 10C). With the cutoff of the median m6A score, the IA samples were divided into high- and low-score groups. The histogram plot showed that Cluster 2 had a higher proportion of the high-score group (Chi-square test, *p* = 0.009; Figure 10D). We also observed that Cluster 1 had a higher proportion of ruptured IAs than Cluster 2 (Chi-square test, *p* = 0.009; Figure 10E). A low m6A score had a larger risk of rupture for IAs (Chi-square test, *p* = 0.022; Figure 10F). The relationships of different m6A subtypes, m6A score groups, and IA rupture status are displayed with a Sanky plot (Figure 10G). Combined with the above result that IAs had a higher m6A score than the controls, the lower m6A score acts as a risk factor for IA formation and IA rupture. Further correlation analysis between the m6A score and immune cells and responses revealed that the m6A score was significantly negatively correlated with the immune characteristics in IAs, implying that inflammation in IAs may also cause the development and even rupture of IAs (Figure 10H).

## Discussion

Posttranscriptional modifications to the chemical composition of RNA molecules may alter their function. The most frequently internally modified base on cellular mRNA is N6 methyladenosine (m6A), which was first discovered on mRNA in the 1970s (Desrosiers et al., 1974). As a result of the lack of methods for mapping the m6A residues on transcripts and the lack of knowledge about the cellular factors that produce and recognize m6A residues, advancements in these fields are very limited. Until the development of genome-wide m6A-mapping methods (Dominissini et al., 2012; Meyer et al., 2012), which revealed the prevalence of m6A throughout the transcriptome, m6A RNA methylation attracted extensive attention and interest among researchers.

Inflammation is the leading factor in the pathogenesis of IAs. Inflammation is triggered by hemodynamic insult, leading to degradation of the extracellular matrix by matrix metalloproteinases (MMPs) and apoptosis of vascular smooth muscle cells (VSMCs). Over time, these processes weaken the arterial wall, causing dilatation, aneurysm development, and ultimately rupture. Although many studies have emphasized that m6A methylation plays critical roles in the development of cardiovascular disorders, little research has been conducted on m6A methylation in IAs. This was the first study to investigate the role of m6A regulators in IAs and revealed the association between m6A methylation and immune characteristics, which could offer reliable directions for future experimental research, provide novel biomarkers, and facilitate the diagnosis and treatment of IAs.

In this study, we curated 24 m6A regulators, among which six genes (*IGF2BP1*, *IGF2BP3*, *YTHDF2*, *ZNF217*, *RBM15*, and *YTHDF3*) were significantly upregulated, and three genes (*YTHDC1*, *FTO*, and *LRPPRC*) were significantly downregulated in IAs and regarded as DEm6A regulators. Biological annotations showed that immune-related pathways (such as complement activation, inflammatory response, and interleukin signaling) and apoptosis were more enriched in IAs than in controls. To further unravel the immune microenvironment in IAs, we performed ssGSEA, which indicated a higher abundance of immune cells and responses and elevated HLA gene expression in IA samples than in controls. The correlation analysis revealed a strong relationship between immune characteristics and DEm6A regulators. We adopted a PCA algorithm and calculated the m6A score of each individual; the results showed that IAs had a lower m6A score than controls. We predicted the targets of DEm6A, screened immune- and apoptosis-related genes, and constructed an immune/apoptosis-related network modified by DEm6A. The m6A sites of six hub targets (CDK1, ASPM, AURKB, BUB1B, MKI67, and TPX2) were predicted with very high confidence using the SRAMP online database. To better understand the contribution of m6A regulators to the pathogenesis of IAs, we established a m6A regulator gene signature using LASSO logistic regression. A diagnostic model with four genes (*LRPPRC*, *YTHDF3*, *IGF2BP1*, and *ZNF217*) was constructed and verified. Two m6A modification subtypes were identified with unsupervised cluster analysis. The biological function analysis indicated that Cluster 2 was enriched in apical junction and TGF beta signaling, whereas Cluster 1 was involved in inflammatory responses. Immune infiltration analysis revealed that Cluster 1 had higher immune activation than Cluster 2. Further study showed that Cluster 1 had a larger proportion of ruptured IAs. These results suggest that m6A modification shapes the IA microenvironment and participates in the formation and rupture of IAs by regulating immune infiltration.

In our enrichment analysis, a series of inflammation-related pathways (such as complement system activation, regulation of neutrophil degranulation, positive regulation of MHC class I biosynthetic process, negative regulation of plasminogen activation, and interleukin signaling) and apoptosis pathways were more enriched in IAs than controls. The complement system comprises component molecules, regulators, and receptors and is one of the first lines of defense against foreign pathogens or stressed cells. Previous studies have demonstrated that complement activation is associated with IA wall degeneration and rupture (Tulamo et al., 2006, 2010). Tulamo et al. (2010) found significantly greater accumulation of complement components in ruptured versus unruptured IAs. Complement deposits with lipids in the outer part of the wall might serve as a marker of a long-term chronic inflammatory process rather than an acute targeted inflammatory reaction. Apart from complement component activation, positive regulation of MHC class I biosynthesis was observed in IAs. We found that the expression levels of most HLA genes (such as *HLA-A*, *HLA-B*, *HLA-C*, *HLA-DRA*, *HLA-DRB6*, and *HLA-F*) were upregulated. The genetic associations between HLA molecules and the pathogenesis of IAs have been widely debated in the last century (Lye and Dyer, 1989; Lye et al., 1989), but little research has been conducted recently. Ostergaard et al. (1986) found that HLA-DR2 antigen frequency is significantly increased (66.7% vs. 29.7%, *p* < 0.01) in IAs compared to healthy controls. However, a later study conducted by Schievink et al. (1988) revealed no significant difference in the HLA-DR2 frequency between IAs and controls, which was further supported by the findings of Lye and Dyer (1989). Our study indicated that the expression levels of most HLA genes were increased. Importantly, these HLA genes strongly correlated with DEm6A regulators, which may provide new experimental directions linking the m6A modification, HLA molecules, and the pathogenesis of IA.

We also evaluated the immune cell infiltration between IAs and controls. The abundance of most immune cells was elevated in IAs. In our next m6A modification subtype analysis, we also observed that Cluster 1, with a larger proportion of ruptured IAs, exhibited higher immune cell infiltration than Cluster 2. These results indicated that Cluster 1 might be the deteriorated stage of IA damaged by inflammation, while Cluster 2 may present with mild vascular injuries; this may help us distinguish the severity of IA in the clinic. The correlation between m6A genes and immune cells revealed that activated CD4 (+) T cells and monocytes are strongly correlated with m6A regulators. CD4 (+) T cells are a key component in cytokine production and act as immune modulators. CD4 (+) T cells are dysregulated in IAs, which may promote inflammatory infiltration (Zhang et al., 2016). When CD4 (+) T cells were cocultured with autologous monocytes, the latter significantly increased IFN-γ and IL-17 production, demonstrating that monocytes could potentially contribute to the altered CD4 (+) T cell composition in IA. CD4 (+) T cells are composed of a heterogeneous group of cells, most commonly T helper 1 (Th1), Th2, Th17, and regulatory T (Treg) cells. m6A regulators can program CD4 (+) T cell differentiation, control their pathogenicity, and promote pathogenesis (Yao et al., 2021; Zhou et al., 2021). Our analysis also indicated the close relationship between m6A genes and immune cells, such as CD4 (+) T cells and monocytes, which may be used to develop targeted immunotherapy in IAs.

In our HALLMARK pathway analysis, the scoring discrepancy of the apoptosis pathway calculated by the GSVA method deserves attention. We observed that apoptosis was more enriched in IAs than in controls. The wall of normal intracranial arteries constitutes a luminal endothelial cell (EC) layer; the internal elastic lamina is composed of elastic fibers; and this is followed by the middle layer consisting of smooth muscle cells (SMCs), elastic lamina, and collagen fibers and is covered by the adventitia layer. The apoptosis of cellular constituents of the vessel wall disrupts the integrity of the vascular wall, leading to aneurysmal dilation, progression, and eventual rupture. An early study discovered apoptosis in 88% of ruptured aneurysms but only 10% of unruptured lesions (Pentimalli et al., 2004). Apoptosis of vascular SMCs can degrade the vascular wall, inducing the development and rupture of IAs (Chalouhi et al., 2012). Considering the important roles of apoptosis and immunity in IAs, we constructed an immune/apoptosis-related target network regulated by m6A modification. Six hub targets (CDK1, ASPM, AURKB, BUB1B, MKI67, and TPX2) were predicted with very high confidence at m6A sites, which may serve as interventional targets.

Subsequently, we constructed a diagnostic model with four identified m6A regulators (IGF2BP1, LRPPRC, YTHDF3, and ZNF217) to predict patients with IAs. Insulin-like growth factor-2 mRNA-binding protein 1 (IGF2BP1), a member of a conserved family of single-stranded RNA-binding proteins, plays an essential role in carcinogenesis (Huang et al., 2018). Recent studies have also focused on its roles in cardiovascular diseases. Silencing IGF2BP1 can protect against ox-LDL-induced lipid accumulation and inflammation by promoting autophagy in macrophages (Liu M. et al., 2022). The knockdown of IGF2BP1 can also greatly reduce macrophage migration (Nguyen et al., 2018). Dong et al. (2021) demonstrated the pivotal role of IGF2BP1 in promoting angiogenesis and atherosclerosis. Given the crucial roles of atherosclerosis and macrophages in the pathogenesis of IAs, we speculated that IGF2BP1 could also be involved in the formation and development of IAs combined with inflammation. Although the roles of *LRPPRC*, *YTHDF3*, and *ZNF217* in cardiovascular diseases have rarely been investigated, the potential interplay between them with immune activity has been identified (Refolo et al., 2019; Zhang et al., 2019). The link between the three m6A methylation regulators and immune infiltration may be a new direction for research on the pathogenesis of IAs, especially when combined with our results, indicating a strong correlation between m6A regulators and immune characteristics.

Our study has certain limitations. First, the small sample size may have caused misinterpretation and bias when conducting unsupervised clustering. Therefore, studies with larger sample sizes must be conducted. Second, although we identified and verified our diagnostic model using internal and external datasets, prospective cohorts are necessary to further investigate the potential diagnostic efficacy in predicting IAs. Third, the analysis results were based on datasets from the GEO database. Although the expression levels of four hub m6A regulators were validated in our blood samples, their expression in IA tissues was not verified because the tissue was too difficult to harvest by surgery with the development of endovascular treatment for IAs. In addition, *in vitro* and *in vivo* experiments should be conducted to verify the bioinformatics results, which is our future research direction. Last, the raw p-value without correct for multiple hypothesis testing was used in our analysis, which could lead to false positive results. Overall, we identified a correlation between m6A regulators and immune characteristics in IAs, which may provide novel biomarkers and ideas for diagnosing and treating IAs.

## Conclusion

We comprehensively analyzed the m6A regulators and immune infiltration characteristics in IAs. An m6A regulator signature (*IGF2BP1*, *LRPPRC*, *YTHDF3*, and *ZNF217*) was established to easily distinguish IAs and healthy individuals, and six hub targets (CDK1, ASPM, AURKB, BUB1B, MKI67, and TPX2) were predicted with very high confidence at m6A sites. Moreover, we identified two distinct m6A subtypes using unsupervised clustering, which may aid in distinguishing the severity of IAs in the clinic.

## Data availability statement

The original contributions presented in this study are included in the article/Supplementary material, further inquiries can be directed to the corresponding author.

## Ethics statement

The studies involving human participants were reviewed and approved by the Institutional Review Board of Xiangya Hospital. The patients/participants provided their written informed consent to participate in this study.

## Author contributions

SL and QZ: conceptualization. SL: methodology, software, formal analysis, investigation, and writing—original draft preparation. QZ: validation. LW: writing—review and editing and visualization. YH and JL: supervision. All authors contributed to the article and approved the submitted version.

## Footnotes

1
http://www.string-db.org

2
http://www.cuilab.cn/sramp

3
http://www.genemania.org/
