# Supplementary material for: Novel insight into m6A regulator-mediated methylation modification patterns and immune characteristics in intracranial aneurysm
Source: Front Aging Neurosci. 2022 Aug 11;14:973258. doi: 10.3389/fnagi.2022.973258 (PMC9404377; doi:10.3389/fnagi.2022.973258)
Supplement: Supplementary file 1 [file Data_Sheet_1.docx]

Supplementary figures


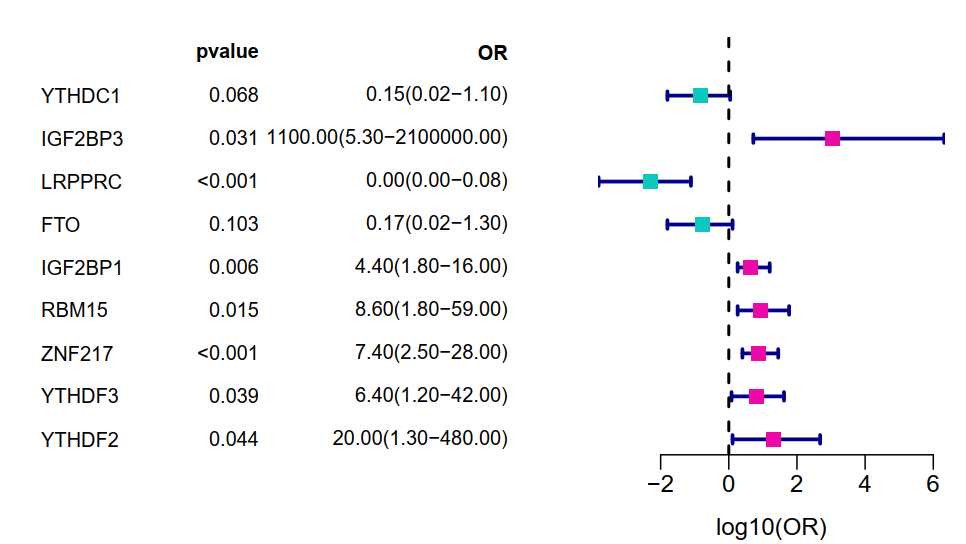


Figure S1. A forest plot showing the results of univariable logistic regression for diagnosis of IA.


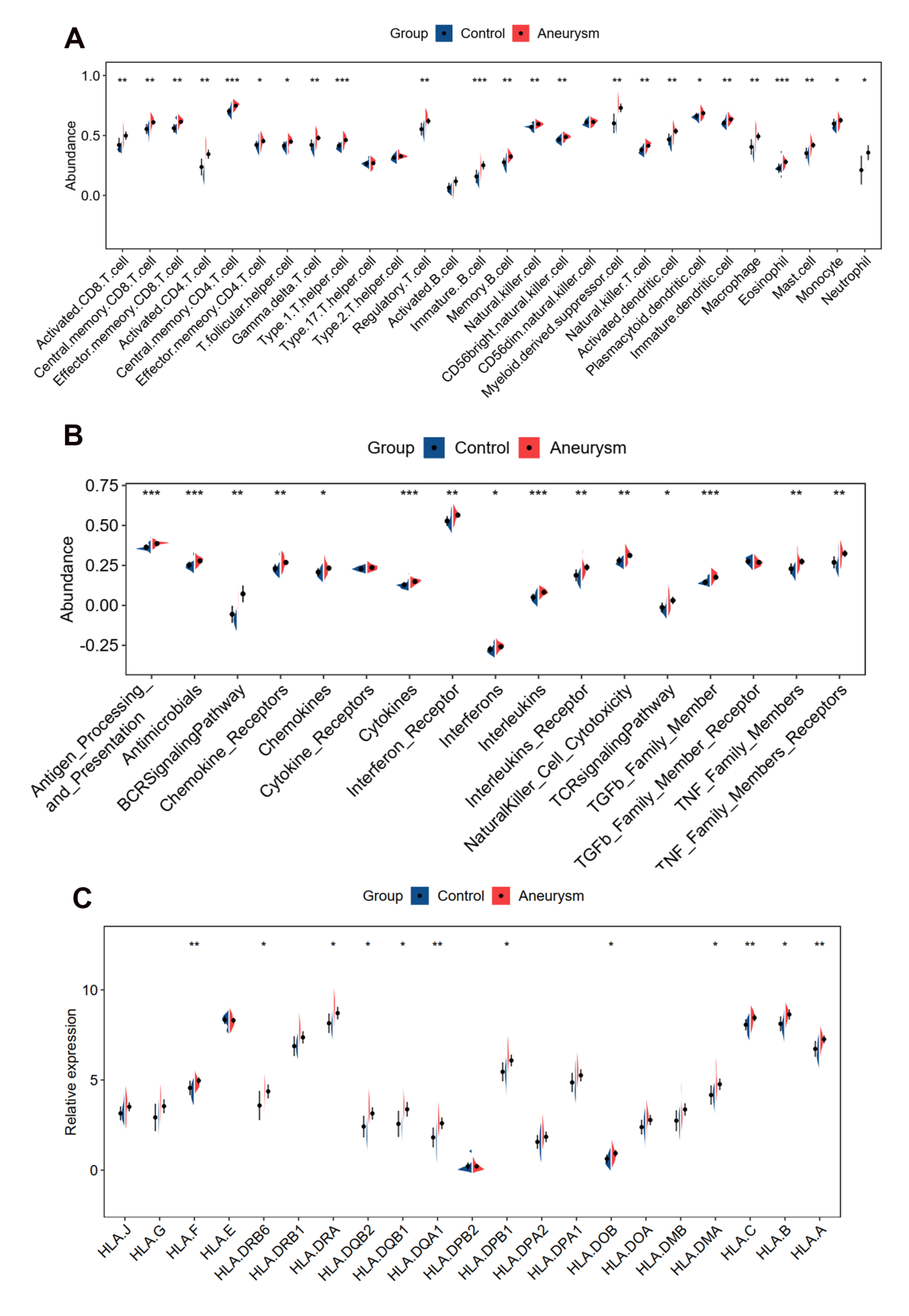


Figure S2. Immune characteristics between IA and controls, including immune cells (A), immune responses (B), and HLA genes (C). Significance level was denoted by *p‐value < .05, **p‐value < .01, ***p‐value < .001.


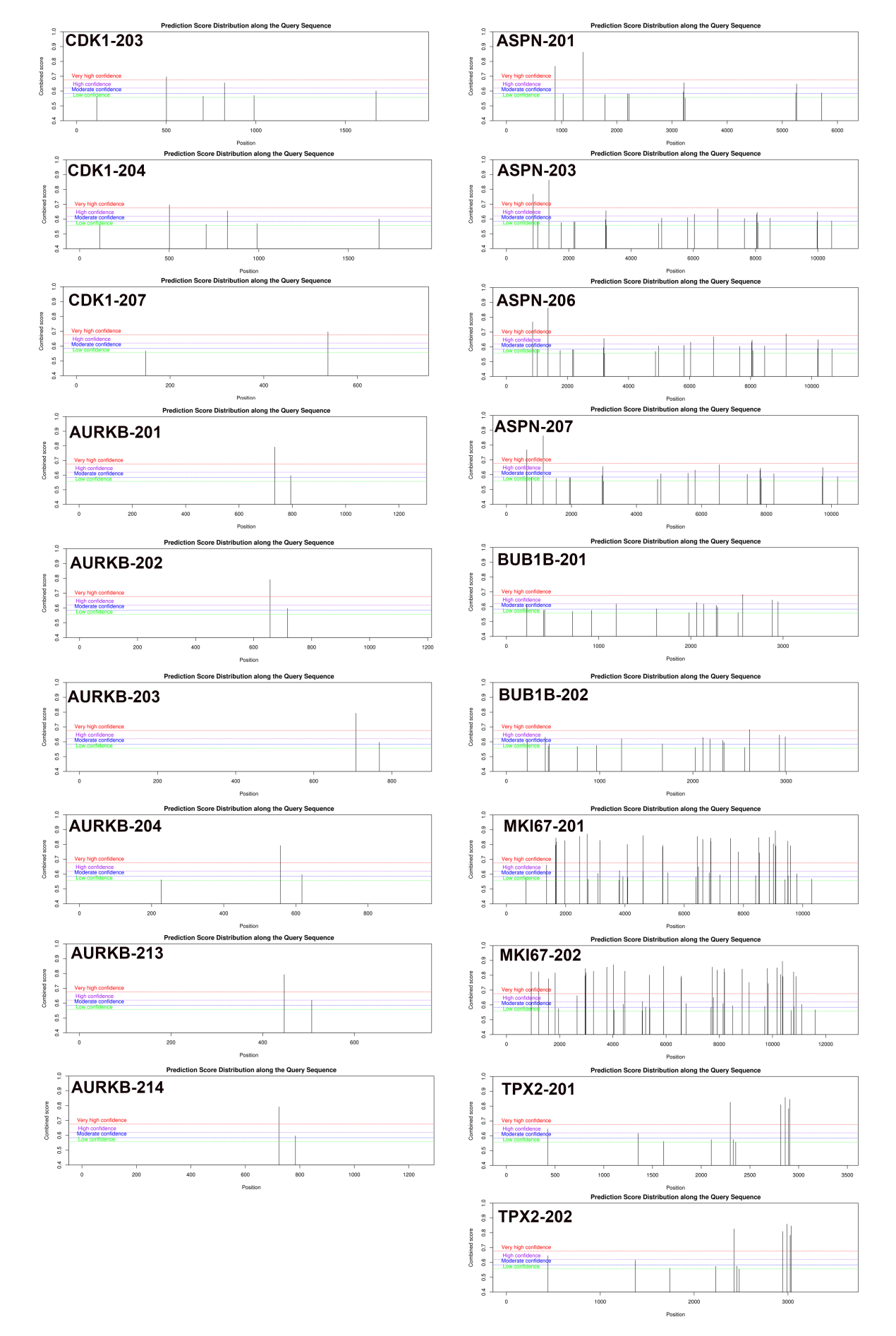


Figure S3. The m6A modification sites with very high confidence of hub targets based on SRAMP online database.


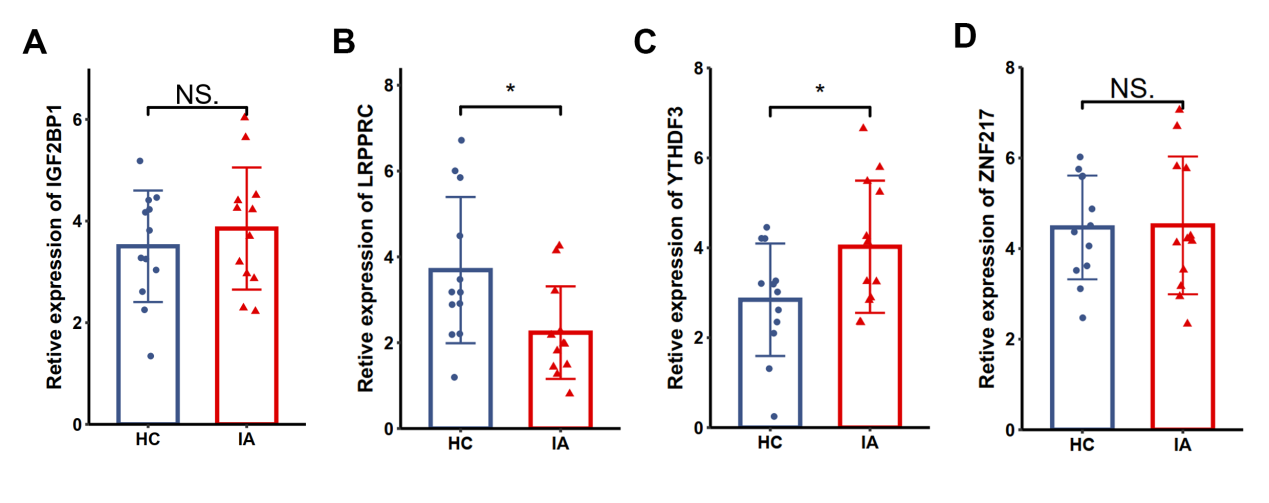


Figure S4. The expression levels of IGFBP1, LRPPRC,YTHDF3, and ZNF217 in our cohort by using qRT-PCR. NS., no significance; *p‐value < .05.


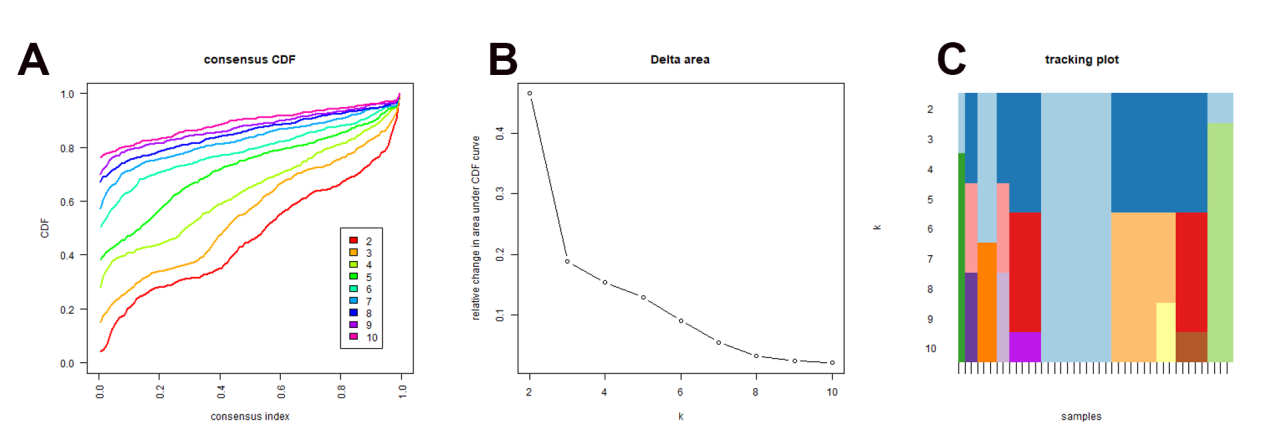


Figure S5. Identification the optimal value of clustering. (A) Consensus clustering of cumulative distribution function (CDF) for k = 2-10. (B) Elbow plot shows relative change in area under CDF curve. (C) The sample distributions under different k = 2-10.


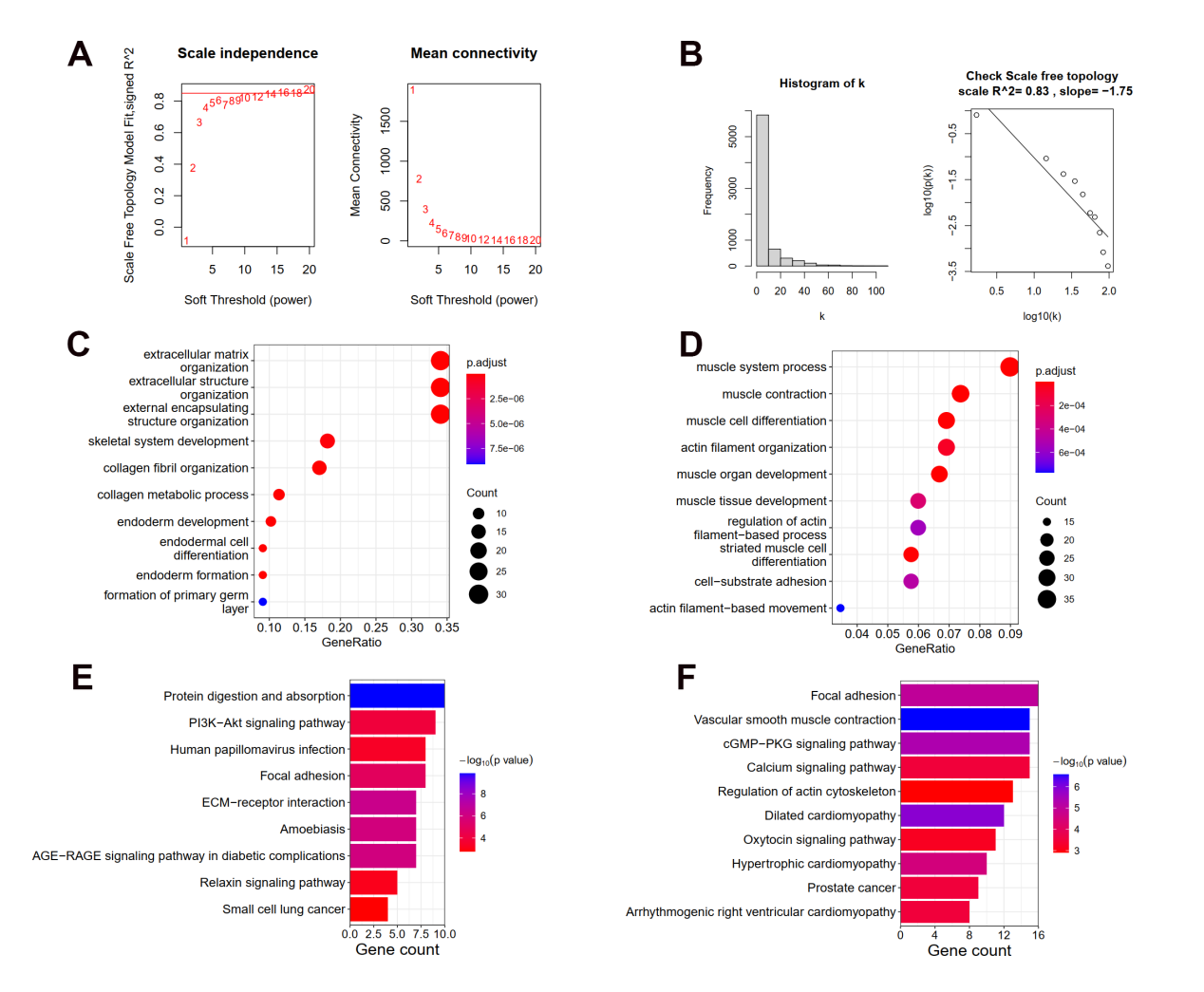


Figure S6. Identification (A) and check (B) of the optimal values of soft threshold to construct a scale free topology based on WGCNA methods. (C) and (E) GO: BP and KEGG analysis for red module key genes. (D) and (F) GO: BP and KEGG analysis for turquoise module key genes.


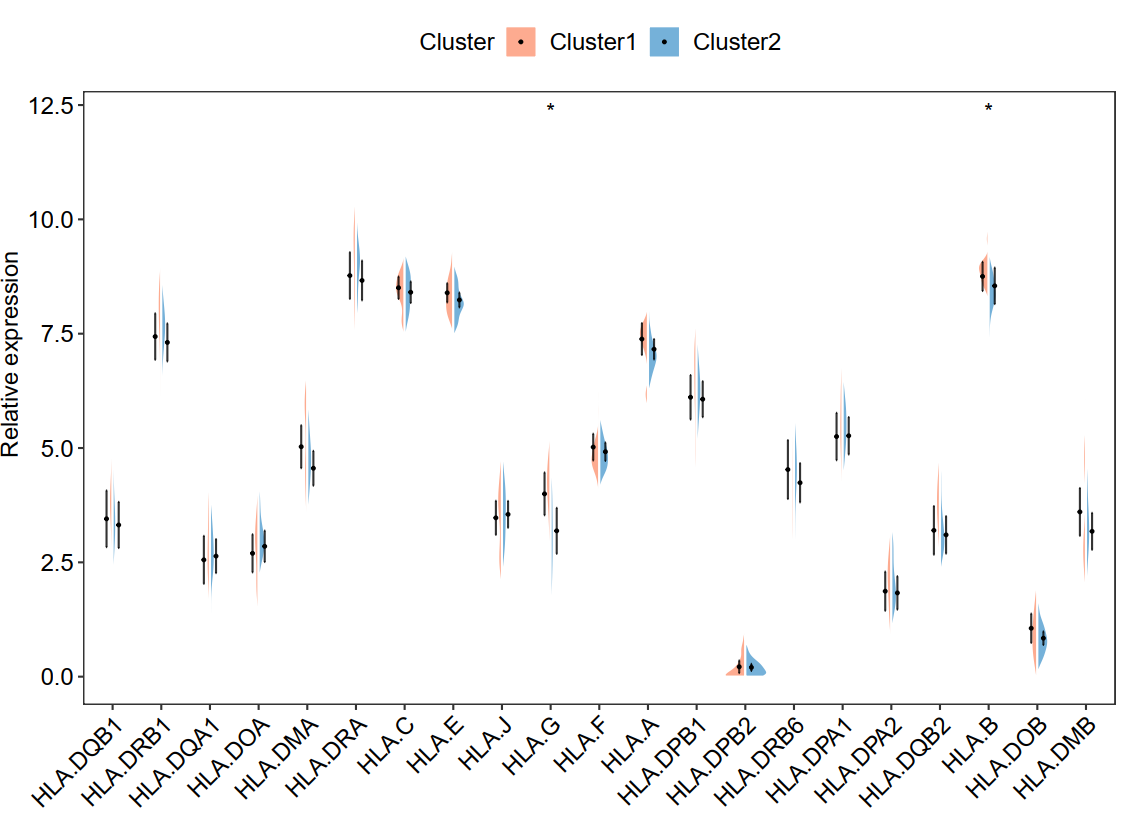


Figure S7. The expression levels of different HLA genes between two subtypes.
